# Surgical treatment for the uncommon bi‐articular fracture of trapezium: A case report

**DOI:** 10.1002/ccr3.7118

**Published:** 2023-06-01

**Authors:** Sadougui Mohammed, Amahtil Mouncef, Bouziane Walid, Hamzaoui Amin, Benhammou Mohammed, Daoudi Abdelkrim

**Affiliations:** ^1^ Orthopedics and Trauma Department Mohammed VI University Hospital Oujda Morocco; ^2^ Faculty of Medicine and Pharmacy Oujda Mohammed First University Oujda Morocco

**Keywords:** carpus, fracture, microscrews, surgery, trapezium bone

## Abstract

Trapezium fracture is a rare condition that goes undetected and exposes to long‐term comorbidities: chronic pain and rhizartrosis. Our work aims to summarize the clinical presentation and improve leading to therapeutic guidelines which are not well established by reporting a case of ORIF with mini‐screws for a displaced fracture of the body of the trapezium with a satisfactory outcome.

## 1 INTRODUCTION

Hand bone fractures represent 18% of all fractures, of which carpal bone fractures constitute 8%. Trapezium bone fractures are uncommon and represent only 4% of all carpal bone fractures,^1^, ^2^ and they are often associated with a fracture of the base of the first metacarpal. These fractures are mostly missed on conventional radiographs with only 18% sensitivity,^3^ leading to chronic pain of the thumb column, loss in grip strength, and rhizarthrosis^4^ if ignored.

The physical findings for this type of fracture are not accurate and usually mimic a scaphoid fracture; therefore, in case of inconclusive radiographs, a CT scan is to be considered.

There are two main types of fractures of the trapezium: the avulsion fractures of the tubercle of the trapezium, which serves as an attachment to the flexor retinaculum; and the body fracture, which is the most frequently found.

We are reporting the case of a 37‐year‐old male presented for a vertical shape displaced fracture of the body of the trapezium, who had undergone an open reduction internal fixation. Through this case, we describe the surgical technique and the functional results, and we also present a brief literature review.

## 2 CASE PRESENTATION

**FIGURE 1:**
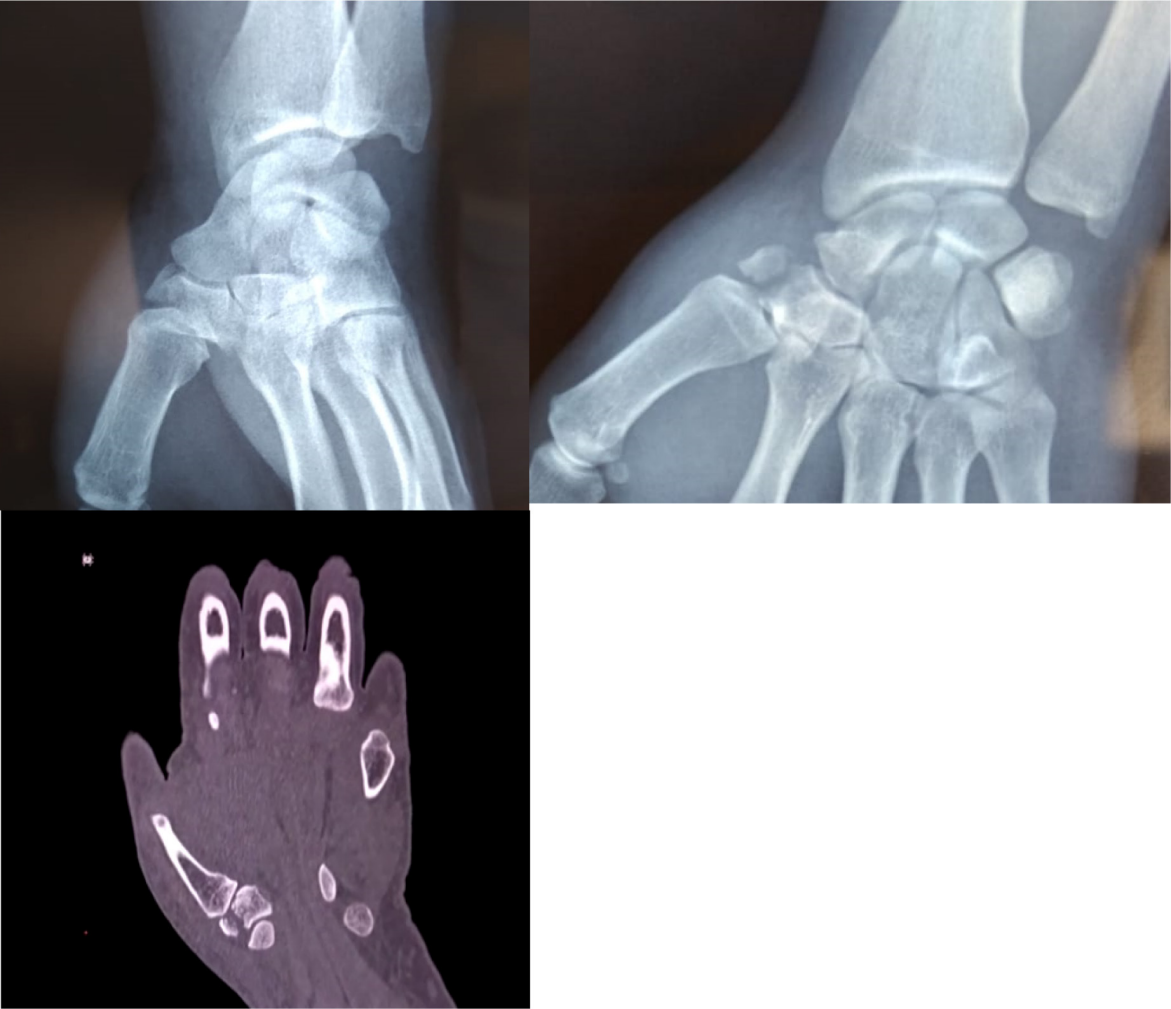
Standard x‐ray and CT scan showing the fracture of the body of the trapezium bone with a vertical line.

**FIGURE 2:**
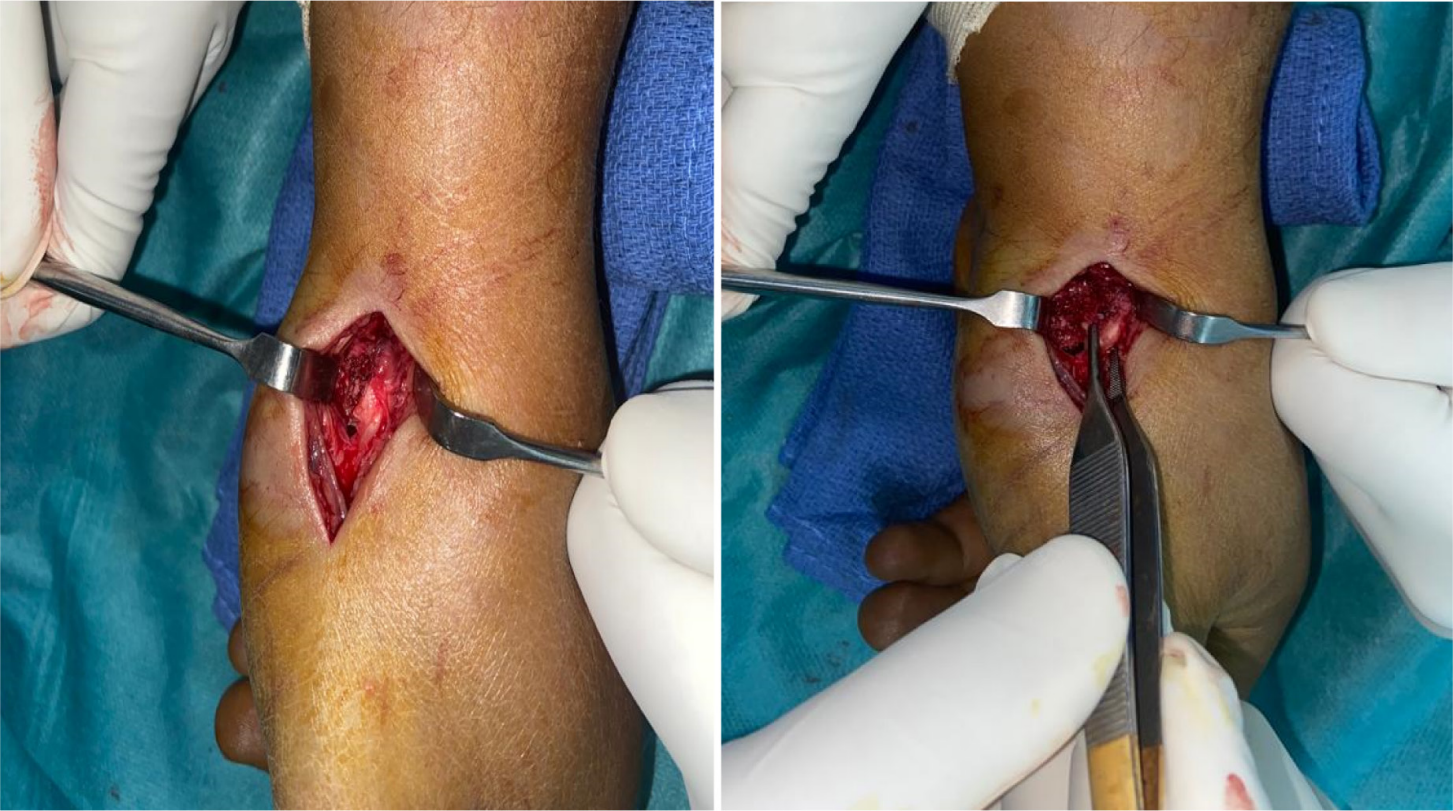
Intraoperative photos showing the approach used and the fracture.

A 37‐year‐old right‐handed male disc jockey, with no notable pathological history, was involved in a traffic accident, and fell from his motorcycle, landing on the palm of his left hand, hyperextended wrist with radial deviation. On admission to the emergency room, the patient presented a swollen wrist with a filled anatomical snuffbox, exquisite pain on compression and rotation maneuver on the thumb column, and palpation of the anatomical snuffbox. The radial artery and other vascular examinations were normal. The hand radiographs showed a vertical fracture of the body of the trapezium bone with a displacement of more than 2 mm, with no associated comminution (Figure 1). The CT scan confirmed the absence of comminution and the absence of any other associated carpal bone lesions. The patient was treated surgically by open reduction and internal fixation with a single microscrew, a dorsal approach was chosen (Figure 2) between extensors pollicis longus and brevis, paying attention to the sensitive branches of the radial nerve and the radial artery, the correct reduction of the articular surface was checked through a longitudinal capsulotomy and fluoroscopic control.

**FIGURE 3:**
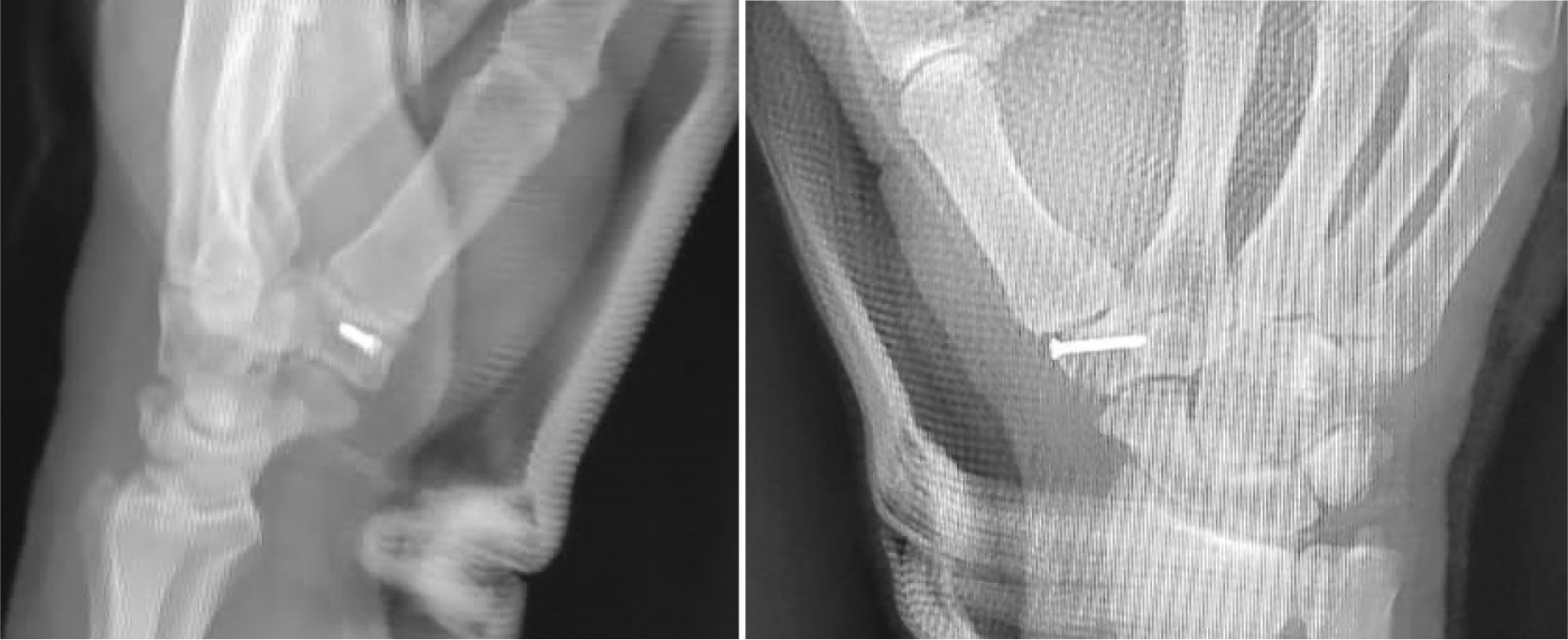
Standard postoperative control radiographs showing good compression of the fracture with the mini screw and the restoration of the articular surfaces.

**FIGURE 4:**
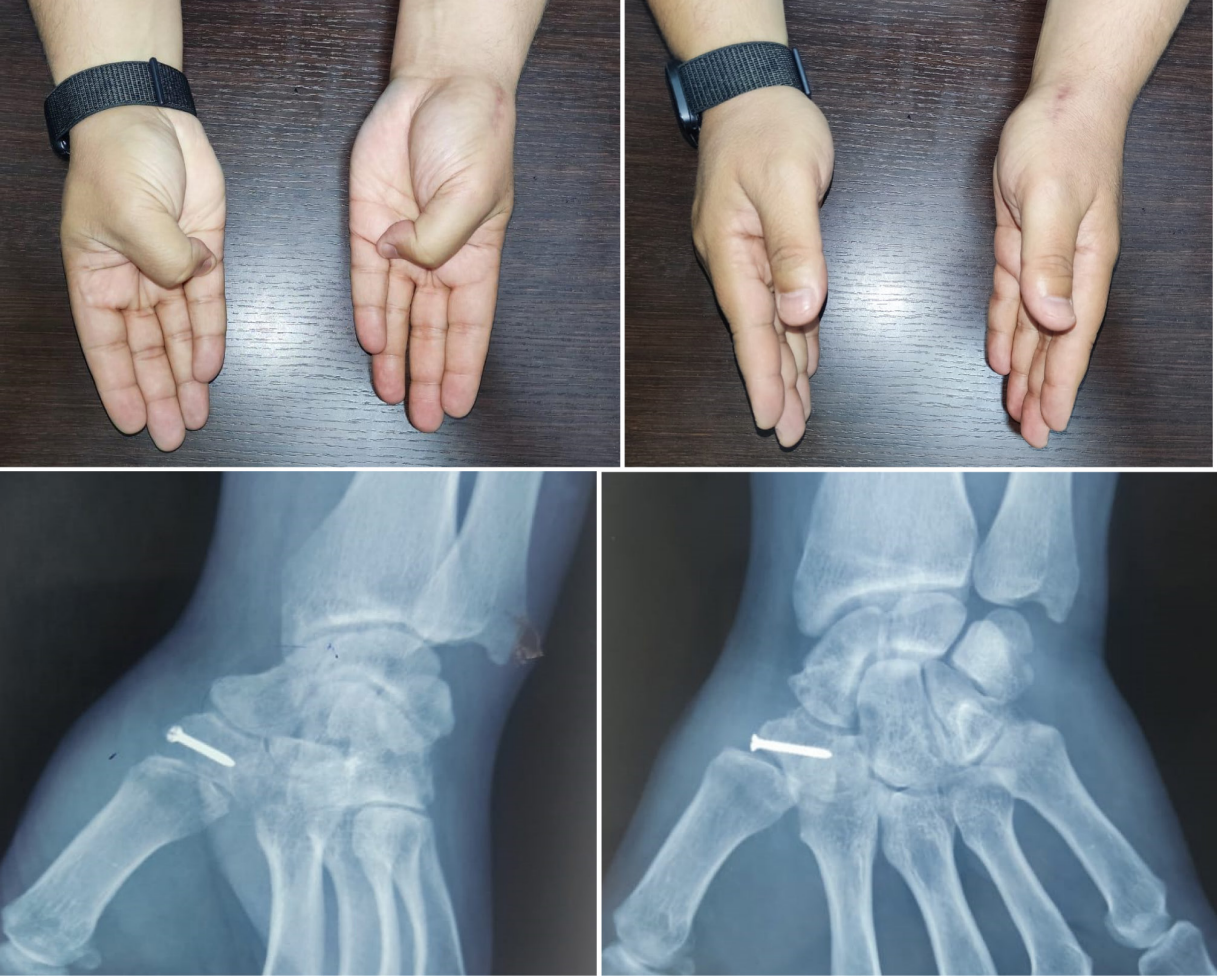
Clinical and radiological results at the end of the sixth week: full restoration of mobility of the thumb column with a kapendji score of 10, and radiography showing complete consolidation of the fracture.

Postoperative radiographs confirmed the accurate reduction of the fracture (Figure 3), and immobilization of the wrist and thumb column in a cast was conducted for 3 week. At cast removal, self‐education was encouraged. The patient regained full mobility of the thumb column and wrist after 5 week (Figure 4), with a return to work at 7 week. The full recovery of grip strength took 9 week. No complications were reported in our patient.

## 3 DISCUSSION

The trapezium bone is the most radial bone of the second row of carpal bones, it articulates with the scaphoid and trapezoid bones proximally and forms a double saddle joint with the base of the first metacarpal distally, providing the thumb a range of mobility in all axes. On its palmar side, the trapezium bone has a central longitudinal crest, which gives an attachment to the flexors' retinaculum, it is called the trapezium ridge.

There are two distinct fractures of the trapezium bone^5^: The first type is the body fracture, the most common one, occurring as a result of a fall onto a hyperextended wrist, with radial deviation and various degrees of abduction of the thumb. The second type is the fracture of the trapezium ridge, which is more unusual and follows a hyperextension of the wrist or a straight impact to the anterior aspect of the trapezium bone, such as a motorcycle handlebars injury. Trapezium ridge fractures are subdivided into two types: Type I is the detachment of the base of the tuber, while Type II is the detachment of the tip. Walker's classification describes five types of trapezium body fractures, depending on the fracture line and the involvement of the articular surfaces, either carpometacarpal (CMC) or scaphotrapezial joint. Type I: fracture with a horizontal line, Type IIa: fracture of the radial tuberosity with involvement of the CMC joint, Type IIb: fracture of the radial tuberosity through the scaphotrapezial joint, Type III: fracture of the ulnar tuberosity, Type IV: fracture with a vertical line, Type V: comminuted fracture.^6^

The clinical presentation of a trapezium fracture is often similar to that of a scaphoid fracture, with swelling and filling of the anatomical snuffbox, ecchymosis, and pain at this level, in some rare cases, the pain is at the anterior aspect, corresponding to a fracture of the trapezium ridge, the pain is aggravated by compression and torsion of the thumb column. Constrained flexion of the wrist, from the position of the wrist in hyperextension, may indicate a fracture of the trapezium tubercle.^5^, ^7^ The neurovascular examination is mandatory. One case of radial artery involvement during a fracture of the trapezium bone has been reported in the literature.^8^

The standard radiographs of the hand front and side can be completed by including a Kapandji incidence, with the wrist pronated at 15°, to eliminate superposition of the trapezium bone with the remaining bones of the carpus or a carpal arch incidence in case of a possible fracture of the trapezial tubercle. However, the sensitivity of standard radiographs for detecting trapezium fractures is still limited. According to a study on 137 wrists with a trapezium fracture diagnosed on CT, the sensitivity of radiographs for detecting this type of fracture was only 18%.^3^ When a trapezium fracture is suspected with inconclusive standard radiographs, it should be followed by CT scan or cone beam imaging, which is less radiating and more accessible than a CT scan and more sensitive than standard radiography.^9^

Orthopedic treatment is proposed for a nondisplaced fracture of the body or the tubercle of the trapezium.^10^ Surgical treatment is recommended for displaced body fractures with more than 2 mm displacement. Open reduction and internal fixation with a miniscrew or Herbert screw allow an anatomical restoration of the articular surface with excellent long‐term functional outcomes.^11^

Some authors suggest excision of the bony fragment in Type‐I fracture of the trapezium tuberosity because of the high incidence of pseudarthrosis and chronic pain.^5^, ^12^ Trapezectomy with or without suspension ligamentoplasty remains a possible indication in elderly patients with preexisting rhizarthrosis, whereas primary arthrodesis remains the only alternative in young, active patients with a highly comminuted fracture.

The purpose of our work is to enrich the literature through the report of our case, especially with the rarity of this type of fracture and the lack of a series of studies that would allow us to reach a well‐coded therapeutic consensus. Surgical treatment of trapezium body fractures allows anatomical restoration of the articular surfaces, thus achieving a good functional outcome and preventing progression to rhizarthrosis and chronic pain.

## 4 CONCLUSION

Isolated trapezium bone fracture is rare, which often goes unnoticed, and this is not sequelae free, it should be systematically searched for before a clinical presentation simulating a scaphoid fracture, especially when radiographs seem to be without abnormalities.

### AUTHOR CONTRIBUTIONS

**Mohammed Sadougui:** Investigation; writing – review and editing. **Mouncef Amahtil:** Investigation; writing – original draft. **Walid Bouzian:** Methodology. **Amine Hamzaoui:** Writing – original draft. **Mohammed Benhammou:** Supervision. **Abdelkrim Daoudi:** Validation.

### FUNDING STATEMENT

This research was not funded.

## CONFLICT OF INTEREST STATEMENT

The authors declare no conflict of interest.

## ETHICS STATEMENT

This is a case report that does not require a formal ethical committee approval. Data were anonymously registered in our database.

## CONSENT

Written informed consent was obtained from the patient for publication of this case report. A copy of the written consent is available for review by the Editor‐in‐Chief of this journal on request.

## PERMISSION TO REPRODUCE MATERIAL FROM OTHER SOURCES

Access to data was approved by the head of the department.

## CLINICAL TRIAL REGISTRATION

This is not an interventional study. We only reported the patient's findings from our database as a case report.

## Data Availability

Not applicable.
